# Biofunctionalization of cardiovascular stents to induce endothelialization: Implications for in- stent thrombosis in diabetes

**DOI:** 10.3389/fphar.2022.982185

**Published:** 2022-10-10

**Authors:** Isra Marei, Blerina Ahmetaj-Shala, Chris R. Triggle

**Affiliations:** ^1^ Department of Pharmacology, Weill Cornell Medicine- Qatar, Doha, Qatar; ^2^ National Heart and Lung Institute, Imperial College London, London, United Kingdom

**Keywords:** in-stent thrombosis, diabetes, cellular dysfunction, endothelialization, endothelial progenitor cells, biofunctionalization, cell capture

## Abstract

Stent thrombosis remains one of the main causes that lead to vascular stent failure in patients undergoing percutaneous coronary intervention (PCI). Type 2 diabetes mellitus is accompanied by endothelial dysfunction and platelet hyperactivity and is associated with suboptimal outcomes following PCI, and an increase in the incidence of late stent thrombosis. Evidence suggests that late stent thrombosis is caused by the delayed and impaired endothelialization of the lumen of the stent. The endothelium has a key role in modulating inflammation and thrombosis and maintaining homeostasis, thus restoring a functional endothelial cell layer is an important target for the prevention of stent thrombosis. Modifications using specific molecules to induce endothelial cell adhesion, proliferation and function can improve stents endothelialization and prevent thrombosis. Blood endothelial progenitor cells (EPCs) represent a potential cell source for the in situ-endothelialization of vascular conduits and stents. We aim in this review to summarize the main biofunctionalization strategies to induce the *in-situ* endothelialization of coronary artery stents using circulating endothelial stem cells.

## Introduction

Cardiovascular diseases are the most prevalent non-communicable diseases worldwide, accounting for 31% of all deaths (WHO, 2017). Coronary artery disease (CAD) is the most common type of cardiovascular disease, causing the majority of cardiovascular-related deaths worldwide (Okrainec et al., 2004). The main cause of CAD is the accumulation of fatty and fibrous materials in the wall of the coronary artery forming an atherosclerotic lesion, which eventually leads to arterial occlusion (Ross, 1993). The growing size of the formed lesion can be sufficient to block the blood flow, however most clinical complications result from thrombus formation. A thrombus obstructs blood flow to the heart muscle leading to myocardial ischemia and infarction (Lusis, 2000).

Percutaneous coronary intervention (PCI) is a non-surgical revascularization technique used to treat obstructive coronary arteries. PCI has become the treatment of choice for CAD. The implantation of intracoronary stents is one of the major PCI techniques used to relive the narrowing of coronary arteries (Smith et al., 2001). Although stenting have improved the acute outcomes of PCI, the long-term outcomes are still hindered by other factors such as age and other comorbidities (Smith et al., 2001). Stent thrombosis and restenosis remain the main causes that lead to vascular stent failure in patients undergoing PCI (Seabra-Gomes, 2006; Chaabane et al., 2013). Stent implantation causes mechanical vascular injury characterized by endothelial denudation and platelet activation, leading to thrombosis and stenosis (Kipshidze et al., 2004; Otsuka et al., 2012; Chaabane et al., 2013).

Evidence suggests that type 2 diabetes mellitus is associated with suboptimal outcomes following PCI or revascularization (Seabra-Gomes, 2006; Bittl, 2015; Koskinas et al., 2016). Type 2 diabetes mellitus is characterized by hyperglycemia and insulin resistance leading to endothelial dysfunction. The abnormal reactivity of diabetic endothelial cells is associated with an increased rate of cardiovascular events (Seabra-Gomes, 2006). In addition, patients with diabetes are at higher risk to develop coronary lesions in stented vessels and are presented with higher rates of completely occlusive restenosis following PCI (Seabra-Gomes, 2006). Diabetes also results in platelet dysfunction and hypo-responsiveness to antiplatelet treatments, increasing the risk of stent thrombosis (Gum et al., 2003; Watala et al., 2004; Angiolillo et al., 2005).

It has been suggested that inducing the rapid endothelialization of stents might improve the outcomes of PCI (Finn et al., 2007). Rapid endothelialization of blood-contacting devices and surfaces is desired due to the anti-thrombotic and anti-adhesive properties of endothelial cells, thus preventing the recruitment and adhesion of platelets and leukocytes to the stented area (Sousa et al., 2003). Establishing a functional endothelial cell layer rapidly after stent implantation might prevent stent thrombosis (Kong et al., 2004). Thus, in this review, we highlight the role of endothelial cells in protecting from stent thrombosis in the context of diabetes, and summarize the main studies that investigated biofunctionalization strategies to induce the *in-situ* endothelialization of coronary artery stents using circulating endothelial stem cells.

### Pathogenesis of stent thrombosis: Role of endothelial cells

Coronary stents are prosthetic cylindrical meshes inserted into the coronary artery using a catheter to relieve the narrowing of the artery and improve blood flow to the heart muscle (Meads et al., 2000). Stents provide a permanent scaffolding for the vessel wall, thus inhibiting the arterial recoil and restenosis associated with plain old balloon angioplasty (Meads et al., 2000; Garcia-Garcia et al., 2006; Seabra-Gomes, 2006). To improve the outcomes of PCI, stents have evolved in terms of design and composition, from bare metal stents (BMS), to drug eluting stents (DES) and bioresorbable vascular scaffolds (BRS). We refer the reader to these reviews on the evolution of stents types, designs and materials (O’Brien and Carroll, 2009; Borhani et al., 2018; Torii et al., 2020; Scafa Udriște et al., 2021).

Stent thrombosis is the occlusion of a coronary artery stent by a thrombus. Standard definitions and classifications of stent thrombosis has been proposed by the Academic Research Consortium (ARC) (Garcia-Garcia et al., 2018). Stent thrombosis is classified into early, late or very late thrombosis according to the elapsed time from stent implantation, and could also be defined according to the degree of certainty as definitive, probable, or silent occlusion (Garcia-Garcia et al., 2018). The reported incidence of stent thrombosis was< 1% for early stent thrombosis (D'Ascenzo et al., 2013), 0.5–1% for late stent thrombosis (D'Ascenzo et al., 2013) and 0.2–0.4% per year for very late stent thrombosis with second generation DES while 2% was reported with 1^st^ generation DES (Biondi-Zoccai et al., 2006). Although stent thrombosis incidence remains low, it constitutes a significant public health issue due to the high number of implanted stents worldwide and the major consequences of thrombotic events (Gori et al., 2019). The mortality caused by stent thrombosis has been reported to be as high as 45% (Biondi-Zoccai et al., 2006). Additionally, stent thrombosis was shown to be accountable for 20% of all myocardial infarction cases following PCI (Gori et al., 2019). Four factors have been identified to influence stent thrombosis including the used device, implantation procedure, patient status, and type of lesion.

The pathophysiological response to stent implantation involves wound healing processes including thrombosis, inflammation, and remodeling (Chaabane et al., 2013). The stenting process leads to a partial or complete denudation of the endothelial cell layer, stretching of the artery, and mechanical vascular injury. This induces platelet activation and adhesion, and the deposition of fibrin on the site of injury. The activated platelets express adhesion molecules, such as P-selectin, which leads to the recruitment of inflammatory cells (Costa and Simon, 2005). The recruited platelets and leukocytes respond by releasing growth factors and cytokines that induce smooth muscle cell proliferation, migration, and deposition of extracellular matrix proteins in the intima of the artery, leading to in-stent restenosis (Chaabane et al., 2013).

Endothelial cells play an important role in protecting from thrombosis and inflammation and maintaining blood fluidity. The release of vasoprotective and thromboresistant agents such as Nitric oxide (NO) and prostacyclin prevents platelet activation and thrombus formation. Von Willebrand factor secretion is also an important factor that modulates platelets adhesion and aggregation under shear conditions (van Hinsbergh, 2012). Additionally, the normal endothelium activates fibrinolysis through the secretion of tissue plasminogen activator; an important mechanism for the resolution of thrombi (Oliver James et al., 2005). Endothelial injury leads to a disturbed production of these protective molecules, and an increase in the expression of adhesion molecules leading to thrombosis, leukocyte recruitment and smooth muscle cell dysregulation (van Hinsbergh, 2012).

The vascular endothelium is also an important interface between the vascular wall and the blood components, and its absence leads to the exposure of the subendothelial elements. The direct interaction of the blood with the subendothelial elements might trigger platelet adhesion leading to thrombosis (Palmaz, 1992). Additionally, implanted stent strut or coating material may induce stent thrombosis (Palmaz, 1992; Jaffer et al., 2015; Georgiadou and Voudris, 2017). It has been determined that the degree of stent coverage with endothelial cells is “the most powerful histological predictor” of stent thrombosis (Finn et al., 2007; Georgiadou and Voudris, 2017). Additionally, the degree of neointima formation following mechanical injury was found to be correlated with the rate of re-endothelialization (Douglas et al., 2013). The delayed stent coverage with endothelial cells in addition to the constant fibrin deposition and inflammation are associated with late and very late stent thrombosis, and the risk is greatly increased in stents with more than 30% uncovered struts (Finn et al., 2007; Claessen et al., 2014).

The stent design and composition are of the main factors that influence stent endothelialization and endothelial cell recovery following PCI (Cornelissen and Vogt, 2019). The surface topography of the stent affects cell adhesion and alignment. It has been shown that a topography resulting in elongated and aligned cells could accelerate the development of a healthy endothelium layer (Claessen et al., 2014). Additionally, the non-physiological nature of the stent material could affect the migration and adhesion of endothelial cells and thus biocompatibility is a key factor in improving endothelialization (Van der Heiden et al., 2013). Endothelialization is also influenced by the thickness of the strut and was shown to be improved in stents with thinner struts (Cornelissen and Vogt, 2019). Additionally, the types of drugs and polymers used in the stent affect cell adhesion and proliferation. While the antiproliferative drugs used in DES reduce neointima formation and in-stent restenosis, they also delay the endothelization of the stent leading to late stent thrombosis (Finn et al., 2007). The incidence of thrombosis in BMS and DES was not shown to be different, and the polymers used in BRS were shown to induce thrombosis (Buchanan et al., 2012). To reduce the occurrence of thrombotic events, dual anti-platelet therapy (aspirin and a P2Y12 inhibitor) is given to patients following PCI (Seabra-Gomes, 2006).

### Stent thrombosis and diabetes

**FIGURE 1:**
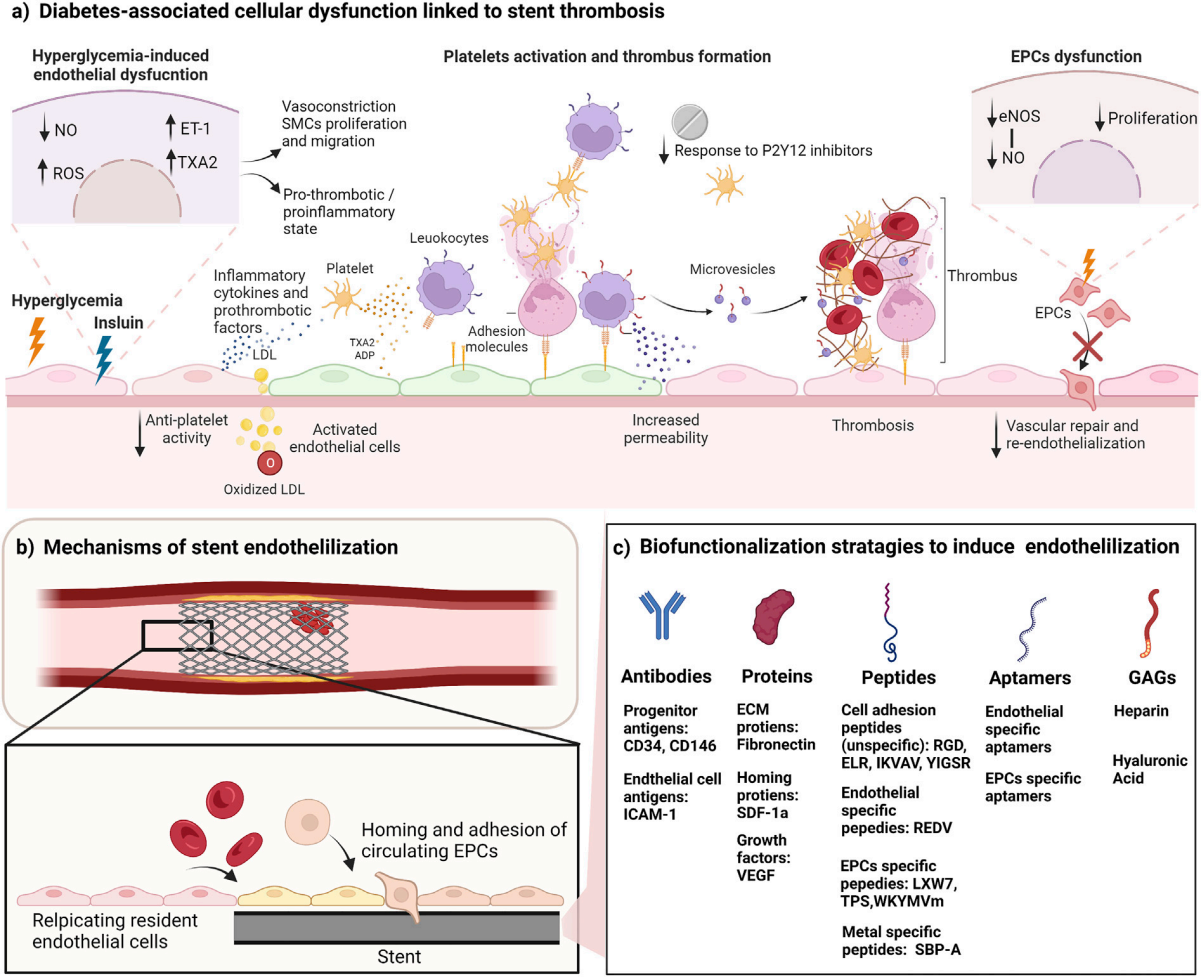
Biofunctionalization of stents to improve endothelialization and reduce thrombosis. **(A)** Cellular dysfunction in diabetes leads to high risk of stent thrombosis. Hyperglycemia results in vascular dysfunction characterized by reduced generation of NO, and induced synthesis of ET-1 and TXA2, resulting in a proinflammatory, pro-thrombotic and vasoconstrictive state. In addition, the chronic activation of endothelial cells by insulin affects the production of vasoprotective and antithrombotic factors. Diabetes also causes platelet hyperactivity, and hypo-responsiveness to anti-platelets drugs. Activated platelets bind to the vascular endothelium directly through adhesion molecules and stimulate an inflammatory response. Platelets also deposit chemokines into the surface of endothelial cells leading to leukocyte recruitment, and platelets can bind to leukocytes that adhere to the endothelial layer. Platelets also can influence endothelial cells by their secretion of vasoactive molecules (such as ADP, serotonin and TXA2) from their granules. The regenerative mechanisms by EPCs are also impaired due to EPCs dysfunction characterized by reduced EPC proliferation, and impaired eNOS and NO production. **(B)** Endothelialization of stents can reduce stent thrombosis. Stent endothelialization happens through 2 mechanisms: resident cell replication and EPC recruitment. Both mechanisms are impaired in diabetes. Targeting these mechanisms can enhance the endothelialization rate. **(C)** Biofunctionalization strategies to promote stent endothelialization. Surface biofunctionalization with mimicry factors aims to induce EPCs mobilization, capture, adhesion, and proliferation. Some of the listed factors also induce the proliferation of resident endothelial cells. NO, nitric oxide; eNOS, endothelial nitric oxide synthase; ROS, reactive oxygen species; TAX2, thromboxane A2; ET-1, endothelin-1; ADP, Adenosine diphosphate; LDL, low density lipoprotein; SMCs, smooth muscle cells; EPCs, endothelial progenitor cells; ICAM-1, intercellular adhesion molecule 1; ECM, extracellular matrix; SDF-1a, stromal cell-derived factor 1; VEGF, vascular endothelial growth factor; GAGs, glycosaminoglycans. Figure was created by BioRender.com.

In diabetes mellitus, patients usually present with platelet dysfunction, hyperactivity or hypo-responsiveness, increasing their risk of stent thrombosis (Yuan and Xu, 2018). Additionally, the vascular endothelium is dysfunctional in response to hyperglycemia, and the proliferation and wound healing responses are impaired in this subgroup of patients (Triggle et al., 2020). Hyperglycemia results in the impairment of endothelial cells, reducing the generation of the vasodilator NO, thus favoring a vasoconstrictive state through the increase in vasoconstrictors and pro-thrombotic mediators, endothelin-1 (ET-1) and thromboxane A2 (TXA2). This imbalance disturbs the vascular tone and results in an increase in smooth muscle proliferation and migration, accompanied by an increased secretion of inflammatory cytokines and prothrombotic factors. The reduction in NO, and the increase of ET-1 and TXA2 induces platelet activation and thrombosis with the potential contribution of an elevated generation of prostacyclin that activates TXA2 receptors (Beckman et al., 2002; Seabra-Gomes, 2006; Vanhoutte and Tang, 2008). These conditions promote thrombus formation (Figure 1A). The incidence of stent thrombosis in patients with diabetes was found to be double that for patients without diabetes (Wiviott et al., 2008). Additionally, insulin was found to play a major role in influencing thrombosis. The chronic activation of endothelial cells by insulin might affect the production of vasoprotective and antithrombotic factors, activating a prothrombotic and proinflammatory status (Wu and Thiagarajan, 1996; Angiolillo et al., 2005). The prothrombotic status in these patients decreases their response to anti-platelet agents. The dysfunctional platelets in patients with diabetes are less sensitive to aspirin increasing their risk of ischemic events (Gum et al., 2003; Watala et al., 2004; Angiolillo et al., 2005). There is also evidence of the negative effect of the common anti-diabetes drug, metformin, on endothelial proliferation on stents releasing mTOR inhibitors, as was shown *in vitro* and in rabbit model (Habib et al., 2013a; Habib et al., 2013b). In terms of the time of occurrence, a meta-analysis of stent thrombosis in patients with and without diabetes have shown that both subgroups had a similar rate of early stent thrombosis following PCI with DES, however, diabetes was associated with an increase in the incidence of late stent thrombosis (Yuan and Xu, 2018).

Given the important role of the endothelium in the protection from thrombosis, re-endothelialization is a key therapeutic target to improve the outcomes of stent implantation in patients with diabetes, and to maintain an antithrombotic and anti-inflammatory status at the site of implantation (Douglas et al., 2013). The gradual endothelialization of stents protects from the thrombotic events, however, this process is slow in BMS, and the drugs used in DES inhibit endothelial cell proliferation and complete coverage. Thus, there is a need for a modulation in the composition of the stents to induce rapid endothelial cell adhesion and proliferation and full stent coverage soon after implantation.

### Stent biofunctionalization to induce endothelialization with circulating endothelial progenitor cells

Endothelialization of stents happens through two main mechanisms: (I) the proliferation and migration of the resident cells at the site of injury, and (II) the homing and adhesion of circulating endothelial progenitor cells (EPCs) (Ong et al., 2005) (Figure 1B). Mature endothelial cells have a low proliferation and replication capacity, thus their participation in the endothelialization process is slow and limited. It is hypothesized that EPCs play a major role in the endothelialization process. EPCs are progenitors that circulate in the blood and have the ability to differentiate to mature endothelial cells and to participate in angiogenesis and neovascularization processes (Medina et al., 2017). Since their discovery by Asahara et al. (1997) in 1997, many attempts have been made to isolate EPCs using varying methods, which resulted in the identification of multiple cell populations that have been categorized under the EPC terminology (Medina et al., 2017). The main identified sub-populations are early EPCs (expressing CD31, CD45 and CD14, and lack expression of CD133) and late EPCs (expressing CD34, CD31 and CD133 and lack expression of the hematopoietic markers CD45, CD14, and CD115) (Tura et al., 2013; O'Neill TJtWamhoff et al., 2005; Zentilin et al., 2006). The late EPCs have been recently recognized to be the “true EPCs” due to their ability to differentiate into a stable mature endothelial phenotype, and to participate directly in the neovascularization process by incorporating into the vasculature (Yoder et al., 2007; Medina et al., 2010a; Keighron et al., 2018). A recent study used single-cell RNA-sequencing analysis (scRNA-seq) to identify specific markers in late EPCs, and found that this subpopulation expressed high levels of bone morphogenetic protein 2 and 4 (BMP 2 and 4) and ephrin B2 (EFNB2) when compared to other types of endothelial cells (Abdelgawad et al., 2021). BMP 2 and 4 were also found to be selectively expressed by late, but not, early EPCs, and to regulate EPC commitment and angiogenic potential (Smadja et al., 2008). Late EPCs and HUVECs share high expression of neuropilin 1 (NRP1) and Vascular endothelial growth factor (VEGF-C) (Abdelgawad et al., 2021), both important factors for the differentiation of endothelial precursors (Cimato et al., 2009; Zhang et al., 2019; Abdelgawad et al., 2021). This expression pattern could be used for the identification and differentiation between subpopulations of EPCs. We refer the reader to these reviews on the detailed differences between these subtypes and their therapeutic potential in many settings including diabetes (Medina et al., 2010b; Yoder, 2012; Pelliccia et al., 2022a; Triggle et al., 2022a).

Biofunctionalization of blood contacting implants and stents using attracting molecules (such as antibodies, proteins, glycosaminoglycan (GAGs), peptides and aptamers) have been proposed to induce endothelialization (Figure 1C). Other delivery approaches have been investigated such as nanoparticles and magnetic molecules. These modifications provide mimicry factors that aim to induce cell capture, adhesion, and proliferation of endothelial progenitors and/or influence their mobilization, taking advantage of their ability to migrate to the site of injury during vascular repair processes. Table 1 summarized some of the recent studies investigating the use of these factors to induce stent endothelialization. We also refer the reader to a comprehensive review on the chemistry aspect of biofunctionalization to incorporate these molecules into the surfaces of medical devices (Spicer et al., 2018).

**TABLE 1:** Summary of the recent studies investigating the use of biomolecules to induce stent endothelialization.

	Stent type/Material	Bioactive molecule	Biofunctionalization strategy	Outcomes	References
Clinical studies	Genous™ EPC capture stent (OrbusNeich, Florida, United States), stainless steel 316L	Murine monoclonal Anti-human CD34+ antibody	Covalently coupled poly-saccharide intermediate matrix coating, immobilized with anti-human CD34+ antibodies.	- Anti CD34 coated stents resulted in rapid endothelialization of stents in murine model (Kutryk and Kuliszewski, 2003). - Clinical studies: TRIAS-HR (Klomp et al., 2011), HEALING (Aoki et al., 2005) and HEALING II (Henricus Eric et al., 2007) showed that the Genous stent was associated with a trend towards increase in target vessel failure. HEALING IIB showed that although statins induced EPC recruitment, combining statin therapy with Genous stent didn’t reduced in-stent restenosis.	(Kutryk and Kuliszewski, 2003; Klomp et al., 2011; Henricus Eric et al., 2007; Klomp et al., 2011; Aoki et al., 2005)
	COMBO bio-engineered stent (OrbusNeich, Florida, United States), stainless steel 316L	Sirolimus and murine monoclonal Anti-human CD34+ antibody	Sirolimus-releasing resorbable polymer matrix (SynBiosys^TM^ urethane-linked multi-block copolymer composed of lactide/ glycolide/ caprolactone/ polyethylenglycol (PEG)) combined with anti CD34+ antibodies.	- COMBO stents were non-inferior to TaxusLiberte™ (REMEDEE randomized study) (Haude et al., 2013), and Xience™ (HARMONEE randomized study) (Saito et al., 2018) - Associated with a trend towards increase in the rates of target vessel failure at 12 months (Saito et al., 2018; Jakobsen et al., 2021). - No difference in 1-year cardiac death when compared to standard DESs. Showed higher rates of target lesion revascularization and target vessel failure (Pelliccia et al., 2022).	(Haude et al., 2013; Saito et al., 2018; Jakobsen et al., 2021; Pelliccia et al., 2022)
	Cobra PzF stent (CeloNova BioSiences, San Antonio, Texas), Cobalt chromium (CoCr)	Fluorinated Polyzene-F (PzF) polymer	Coated with a thin nano-layer of fluorinated Polyzene-F (PzF) polymer, and a layer of poly (bis [trifluoroethoxy]phosphazene).	- PzF previously showed reduced intimal hyperplasia, anti- thrombotic, and anti-inflammatory properties (Koppara et al., 2016) and had superior healing when compared to bioabsorbable polymer DES in porcine and rabbit models (Hiroyuki et al., 2019). - Clinical studies: 1-year follow up showed that the stent performance is satisfactory and confirmed clinical efficacy and safety (Maillard et al., 2021). - 5 years follow up showed low incidence of major adverse clinical events, with no reported stent thrombosis throughout the 5 years. Target vessel failure increased form 11.5% at 9 months to 17.4% at 5 years (Cutlip et al., 2022).	(Koppara et al., 2016; Hiroyuki et al., 2019; Maillard et al., 2021; Cutlip et al., 2022; Cornelissen et al., 2022)
*In vivo* studies	CoCr	A homing peptide for endothelial colony forming cell (WKYMVm)	Stents were coated with dopamine, and the peptide was conjugated to dopamine using N-hydroxysuccinimide (NHS) and 1-Ethyl-3-(3-dimethylaminopropyl) carbodiimide (EDC) to activate the carboxyl group of the peptide.	*- In vitro:* The modified stent improved the proliferation of HUVECs at day 7 of culture in comparison to BMS. *- In vivo:* peptide delivery to vessels was studied in rabbit iliac arteries, and peptide coating was observed up to 7 days, and diminished gradually.	(Bae et al., 2020)
	Stainless Steel	Murine monoclonal antihuman endoglin antibody	Commercially available stents: murine monoclonal antihuman endoglin antibody (ENDs) (Beijing Lepu Medical Technology limited corporation, China), in comparison to sirolimus eluting stents (SESs) (Johnson & Johnson, United States), and BMS (Abbott, United States).	Animal model: juvenile pigs. Findings: Mean neointima area and percent area stenosis were lower in ENDs and SESs when compared to BMSs at 14 days of implantation. Endothelial coverage of ENDs was significantly higher than that of SESs and BMSs at days 7 and 14, indicating induced endothelialization.	(Cui et al., 2014)
			Stainless steel stents coated with murine monoclonal ENDs and CD34s (Beijing Lepu Medical Technology limited corporation, China), and SESs (Johnson & Johnson, United States).	Animal model: pigs. Findings: mean neointima area and ENDs, SESs and CD34s were lower in ENDs, SESs and CD34s when compared to DES at day 14 of implantation. Endothelial coverage was induced in ENDs and CD34 when compared to SESs and BMSs at days 7 and 14.	(Cui et al., 2015)
	CoCr	Anti CD146 antibody and silicone (si) nanofilaments	Polished surfaces were coated with si nanofilments. Surfaces were treated with O2 plasma, followed by immersion in toluene dissolved in 3-aminopropyltriethoxysilane (APTES) to introduce amine groups. Antibodies were immobilized in the presence EDC and NHS.	- *In vitro*: both si nanofilaments and CD146 induced EPCs and MSCs capture under dynamic conditions (15 dyne/cm^2^) in a perfusion pump system. Cell adhesion and spreading was improved on modified surfaces. - *In vivo*: stents were implanted into porcine coronary arteries for 1 week, and showed enhanced endothelial coverage in stents coated with both si nanofilaments and CD146 antibody. The modified stents reduced restenosis when compared to BMS.	(Park et al., 2020)
	Stainless steel 316L	Recombinant antibody fragments (scFv) specific for vascular endothelial growth factor receptor-2 (VEGFR2)	Surfaces were coated with titanium precursor followed by functionalization with amino groups and immobilization of oxidized glycosylated scFv molecules.	*- In vitro*: The modification didn’t affect the metabolic activity or induce cytotoxicity of HUVECs. Adhesion of HUVECs was increased on VEGFR2 scFv surfaces. - *In vivo*: stents were implanted into porcine arteries for 5 and 30-days. There was no evidence of restenosis, thrombosis, or myocardial infarction at both time points. Stent coverage was significantly higher in modified stents when compared to BMS at 5 days. No significant difference was detected at 30 days. Histological sections showed coverage with a cell layer (80 μm) by day 30.	(Wawrzyńska et al., 2020)
	Nitinol	RGD peptide and CXCL1	Stents were coated with star-shaped polyethylenglycole (PEG), followed by immobilization of RGD alone or RGD/CXCL1.	- *In vitro*: increased adhesion of EOC and HUVEC to RGD and RGD/CXCL1 surfaces compared to BMS and star-PEG modified surfaces. Smooth muscle cells (SMCs) proliferation was not affected in RGD/CXCL1 and was reduced in star-PEG surfaces. - *In vivo*: stents were implanted in apoE-/- mice for one week, and showed reduced stenosis and thrombosis in RGD and RGD/CXCL1 stents. Star-PEG stents resulted in induced thrombosis. Endothelialization was increased in RGD/CXCL1 stents.	(Simsekyilmaz et al., 2016)
	Stainless Steel	Vascular endothelial cadherin (VE-Cad) antibody	VE-Cad antibodies were immobilized on stainless steal stents grafted with sulfonamide zwitterionic and acrylic acid.	*- In vitro:* the modified stent with the co-polymer didn’t cause blood cell or platelet adhesion or activation. Stents containing VE-Cad antibody resulted in induced EPCs adhesion and coverage, while small numbers were adhered to BMS and stents with the co-polymer alone. - *In vivo*: stents were implanted into rabbit carotid artery, and showed no signs of thrombosis or stenosis following 1 month of implantation. Modified stents were completely covered with endothelial cells.	(Chen et al., 2017)
	Titanium (Ti)	heparin/poly L-lysine nanoparticles	The nanoparticles were immobilized into dopamine coated Ti surfaces. Ti disks were coated with dopamine (2 mg/ml in Tris buffer, pH 8.5) for 12 h, followed by sonication in water. The process was repeated three times to coat with three layers, followed by incubation with nanoparticle suspension at 37°C for 24 h.	Ti modified samples were implanted into dog femoral arteries for 4 weeks. Ti surfaces showed severe thrombus formation and thick neo-intimal formation, whereas Ti modified surfaces showed no thrombosis or neointimal thickening. The Ti modified surfaces were also covered with a confluent layer of endothelial cells.	(Liu et al., 2014)
	Stainless Steel	Vildagliptin	Electrospinning of poly (D,L)-lactide-co-glycolide (PLGA), in combination with vildagliptin (240 mg/40 mg or 260 mg/20 mg) and hexafluoro isopropanol (HFIP). The nanofibrous sheets were mounted on commercially available BMSs (Gazelle BMS, Biosensors International, Switzerland) followed by vacuum drying.	*- In vitro:* Migration of HUVECs in transwells was enhanced in presence of vildagliptin eluents. *- In vivo: P*ure PLGA stents and vildagliptin eluting stents (low and high dose loading) were implanted into mechanically-denuded abdominal aorta of alloxan-induced-diabetic rabbits. Vildagliptin stents resulted in superior coverage with endothelial cells following 2 months of implantation when compared to pure PLGA stents. Nanofibrous stents induced the alignment of cells and insured cell-cell contact, unlike the pure PGLA stents. Endothelium- dependent vasodilation response to acetylcholine was higher in vildagliptin stents.	(Lee et al., 2019)
*In vitro* studies	CoCr	Elastin-like recombinamers (ELR) genetically modified with an REDV sequence	Plasma activation and etching using sodium hydroxide (NaOH) followed by silanization with 3-chloropropyltriethoxysilane and functionalized with the ELR	HUVEC cell adhesion response time was directly correlated to the amount of immobilized ELR on the surface. Surfaces activated with NaOH showed better adhesion and spreading of HUVECs.	(Castellanos et al., 2015)
	Stainless Steel 316L	Phage identified SUS316L-binding peptide (SBP-A, VQHNTKYSVVIR), followed by anti ICAM-1 antibody modification	The SBP-A peptide was used as a linker to immobilize ICAM-1 antibody. N-terminal streptavidin-modified anti-ICAM antibody was added to SBP-A-modified SUS316L disks.	The identified peptide (SBP-A) was not toxic to HUVECs. The described modification with SBP-A and anti ICAM-1 antibody influenced HUVECs adhesion and showed higher selectivity to HUVECs over SMCs.	(Sakaguchi-Mikami et al., 2020)
	CoCr	Endothelial specific oligonucleotide: 5’ -GGG AGC TCA GAA TAA ACG CTC AAC AAC CCG TCA ACG AAC CGG AGT GTG GCA GGT TCG ACA TGA GGC CCG GAT C-3’	Aminosilanization using (3-Aminopropyl)triethoxysilane (APTES), followed by immobilization of of 3'-thiol modified oligonucliotide.	Porcine EPCs showed enhanced adhesion to modified surfaces and were able to proliferate and reached confluence in 4 days of culture.	(Barsotti et al., 2015)
	Ti	Ti oxide (TiO_2_) nanotubes and fibronectin	TiO_2_ surfaces were anodized to create TiO_2_ nanotubes. Fibronectin was immobilized on TiO_2_ nanotubes using polydopamine	Fibronectin functionalized TiO_2_ nanotubes enhanced the adhesion, spreading, proliferation and secretion of nitric oxide and prostacyclin in HUVECs. The nanotube size had an inverse relationship with cytocompatibility.	(Jin et al., 2018)
	Ti	Ti nanotubes	Anodic oxidation	Ti nanotubes induced VEGF production by macrophages. Also, they inhibited glycolysis of macrophages by activating AMPK signaling, leading to reduced macrophage release of inflammatory factors and induced polarization, accelerating endothelialization.	(Yu et al., 2021)
	Nitinol	Semi-interpenetrating network (IPN) hydrogel consisting of Polyacrylamide (PAAm), polymethyl methacrylate (PMMA), polyurethane and polydopamine	Cast molding of stents in semi IPN hydrogel through free radical polymerization	Induced adhesion, proliferation, and migration of HUVECs. Reduced adhesion and proliferation of SMCs.	(Obiweluozor et al., 2019)
	Stainless steel 316L	Recombinant antibody fragments (scFv)	Incorporating hydroxyl groups through coating with titania, followed by silanization using APTES, and immobilization of glycosylated scFv.	The modification was nontoxic to the EPC line 55.1 (HucPEC-55.1) and maintained their viability on modified steal.	(Foerster et al., 2016)
	bio-absorbable magnesium alloy MgZnYNd	Arginine-leucine based poly (ester urea urethane)s (Arg-Leu-PEUUs) in comparison to poly (glycolide-co-lactide) (PLGA) coating	Spinning coating of disks with the polymers (Arg-Leu-PEUU in N,N-Dimethylformamide (DMF) or PLGA in Dichloromethane CH_2_Cl_2_) followed by solvent evaporation and heating.	Enhanced HUVECs viability, which was proportionally related to Arg ratio. HUVECs increased NO production. Viability of SMCs was not affected by the peptide.	(Liu et al., 2017; Liu et al., 2017)
	Ti	Heparin-VEGF-fibronectin	Layer-by-layer coating	The modification resulted in reduced platelet adhesion and aggregation and prolonged partial thrombin and prothrombin time, compared to unmodified Ti. HUVECs adhesion and proliferation were induced on modified surfaces	(Wang et al., 2013)
	PEG-diacrylate (PEGDA) hydrogel	REDV-containing peptides	Peptides that target α4β1 and α5β1 were coupled to PEGDA hydrogel using these combinations: RGDS+ REDV CRRETAWAC(cyclic)+REDV, P_RGDS+ KSSP_REDV, P_RGDS+ P_RDEV P_RGDS+ P_REDV	CRRETAWAC(cyclic)+REDV, P_RGDS+KSSP_REDV, and P_RGDS+P_REDV induced late EPCs capture under dynamic conditions in a parallel plate flow chamber system at 20 s^–1^, and resulted in high tether percentages and velocity fluctuation	(Tian et al., 2022)

To this date, the main clinically applied biofunctionalization strategy to induce EPCs capture and stent endothelization is the use of monoclonal antibodies against CD34, represented by the Genous™ EPC capture stent and the COMBO bio-engineered stent (OrbusNeich, Florida, United States) (Klomp et al., 2009; Tomasevic et al., 2019) (Table 1). CD34 biofunctionalized stents showed a great promise in early *in vivo* models, as they resulted in the rapid endothelialization of stents in a murine model (Kutryk and Kuliszewski, 2003). Also, early *ex-vivo* and clinical studies showed the rapid endothelialization of BMS (Larsen et al., 2012) and DES (Granada et al., 2010; Nakazawa et al., 2010), and for that it was hypothesized that these stents will protect from stent thrombosis. Despite their initial promise, recent clinical studies comparing the performance of the Genous™ EPC capture stent with DES didn’t show superior results in terms of their protection from lumen loss and restenosis. Studies including the TRIAS-HR (71), HEALING and HEALING II (Duckers et al., 2007) showed that the Genous stent was associated with a trend towards increase in target vessel failure. In light of these findings, it was thought that combining the CD34 capture antibody with an anti-proliferative drug will improve these outcomes, thus the novel COMBO bio-engineered stent was developed.

The COMBO bio-engineered stent (OrbusNeich, Florida, United States), is a new generation DES which contains a sirolimus-releasing resorbable polymer matrix to reduce restenosis, in addition to the CD34 coating to induce endothelization. Although comparative clinical trials have shown that COMBO stents were non-inferior to other DESs including TaxusLiberte™ (REMEDEE randomized study) (Haude et al., 2013), and Xience™ (HARMONEE randomized study) (Saito et al., 2018), the COMBO stents were associated with a trend towards increase in the rates of target vessel failure at 12 months (Saito et al., 2018; Jakobsen et al., 2021). Additionally, a recent systematic review including a total of 3961 patients and comparing the COMBO EPC-capturing DES against standard DES from 4 randomized controlled trials, showed no difference in 1-year cardiac death when compared to standard DESs. However, COMBO stent was associated with higher rates of target lesion revascularization and target vessel failure (Pelliccia et al., 2022b). Thus the benefit of these stents in inducing rapid endothelization should be weighed against the possible risk of induced hyperplastic reactions and their consequences (Pelliccia et al., 2022b).

The use of CD34 antibody to capture EPCs has also been proposed for other medical devices, vascular grafts and tissue engineering scaffolds. Nevertheless, because CD34 is not specific to EPCs, it has been suggested that other CD34 positive cells in the blood will compete with EPCs to adhere to the immobilized antibody (Sidney et al., 2014). This could be a contributing factor to the hyperplasia observed in the CD34 biofunctionalized stents. The use of other antibodies against CD133, and VE-Cadherin, amongst other antigens, have been reported to influence EPCs capture, however these stents have met mixed success *in vivo* (Sedaghat et al., 2013; Van der Heiden et al., 2013). Therefore, there is a need to incorporate other specific bioactive moieties to induce the specific recruitment of EPCs without inducing hyperplasia and restenosis.

Other EPC capturing strategies have been investigated *in vitro* and *in vivo,* however these approaches are yet to be validated and translated to clinical use (Table 1 respectively). Growth factors such as vascular endothelial growth factor (VEGF) have been used to induce EPCs adhesion and growth (Van der Heiden et al., 2013). Mobilization of stem cells using chemokines such as stromal cell derived factor 1a (SDF-1a) have also been investigated (Zhang et al., 2011). However, these factors are not specific to EPCs and might result in similar outcomes to what has been observed in CD34 coated stents.

A more specific approach to capture EPCs is the use of specific short peptide ligands and aptamers. These ligands provide an advantage over large biomolecules, because controlling the configuration and folding of large biomolecules is challenging during the biofunctionalization process. The literature describes the use of peptides with different specificities: (i) metal-binding peptides, (ii) non-specific cell adhesion peptides, (iii) endothelial cell-specific peptides and (iv) EPC-specific peptides. Metal-binding peptides are used as linkers to allow further modifications of the stent surface. Examples include the stainless-steel specific peptide SBP-A (Sakaguchi-Mikami et al., 2020).

One of the commonly used peptides that has shown enhanced cell biocompatibility is RGD (Arginyl-glycyl-aspartic acid) peptide, which is the principle ligand responsible for cell binding to the ECM (Bellis, 2011). Other peptides have been investigated such as the laminin derived sequences IKVAV (isoleucine-lysine-valine-alanine-valine) and YIGSR (Tyrosine-Isoleucine-Glycine-Serine-Arginine) (Massia and Hubbell, 1991; Grant et al., 1992). These peptides enhance the non-specific adhesion of cells to biofunctionalized surfaces. Peptides targeting endothelial cells have also been investigated, including REDV (Arginine-Glutamate-Aspartate-Valine) (Hubbell et al., 1991). Specific peptides to EPCs have been identified such as the disulfide cyclic octa-peptide (cGRGDdvc, also known as LXW7) (Hao et al., 2017), TPS (Threonine-Proline-Serine-Leucine-Glutamate-Glutamine-Arginine-Threonine-Valine-Tyrosine-Alanine-Lysine) (Veleva et al., 2007), and WKYMVm (Trp-Lys-Tyr-Met-Val-D-Met) (Bae et al., 2020a). These peptides interact with the integrins -which are adhesion receptors on the cells - and activate them, resulting in enhanced cell adhesion and binding. EPCs specific aptamers or oligonucleotides have been also tested (Barsotti et al., 2015). These bioactive molecules hold a great promise for the biofunctionalization of stents due to their specificity and ease of incorporation.

### Challenges facing stent endothelialization with EPCs

One of the main challenges facing the *in situ* endothelialization with circulating EPCs is their low numbers in the blood (Yoder, 2012). These numbers were also shown to be reduced in disease states such as diabetes. Thus, strategies to boost the numbers of EPCs might be required. One example is the use of pharmacological induction using agents with known effects on EPCs such as statins. It was observed during the HEALING IIB study that statin therapy has increased the numbers of EPCs by 5.6-fold, and that the combination of statin therapy with EPC capturing stents resulted in optimal coverage of the stents (den Dekker et al., 2011). EPCs numbers could be boosted by other strategies such as combining more than one capturing molecule or incorporating chemokine or growth-factor-releasing nanoparticles within the coating of the stent. Additionally, local or systematic injection of autologous EPCs could help to boost the endothelialization of the stent.

Another limitation of stent endothelialization with EPCs is the variability in the intrinsic regenerative potential between patients, which might be affected by diabetes, cardiovascular diseases or other comorbidities (Emmert and Hoerstrup, 2016). This is important to consider particularly because the whole concept of *in situ* endothelialization depends on the intrinsic regenerative potential, and any impairment of this potential will affect the rate of endothelialization (Stassen et al., 2017). It was shown that EPCs function and regenerative ability is impaired in diabetes (Triggle et al., 2022b). This, in addition to vascular endothelial dysfunction, reduces the potential of stent coverage. Thus, enhancing endothelial and EPC function in these patients should be a target to improve endothelialization, in combination with stent biofunctionalization. Antidiabetic drugs with endothelial and cardioprotective effects (such as vildagliptin) (Lee et al., 2019) could be investigated in combination with the biofunctionalized stents.

In conclusion, stent endothelialization represents a potential target to reduce in-stent thrombosis following PCI. Specific biofunctionalization of stents is required to induce endothelialization without evoking restenosis. Targeting EPC and endothelial dysfunction in diabetes are key strategies to aid in the endothelialization process.
